# Multimorbidity Patterns in Hospitalized Older Patients: Associations among Chronic Diseases and Geriatric Syndromes

**DOI:** 10.1371/journal.pone.0132909

**Published:** 2015-07-24

**Authors:** Mercedes Clerencia-Sierra, Amaia Calderón-Larrañaga, Nicolás Martínez-Velilla, Itziar Vergara-Mitxeltorena, Pablo Aldaz-Herce, Beatriz Poblador-Plou, Mónica Machón-Sobrado, Nerea Egüés-Olazabal, Gabor Abellán-van Kan, Alexandra Prados-Torres

**Affiliations:** 1 Unit of Social and Clinical Assessment (UVSS), Miguel Servet University Hospital, Zaragoza, Spain; 2 EpiChron Research Group on Chronic Diseases, Aragon Health Sciences Institute (IACS), IIS Aragón, Miguel Servet University Hospital, Zaragoza, Spain; 3 Research Network for Health Services in Chronic Diseases (REDISSEC), Madrid, Spain; 4 Geriatric Service, Hospital Complex of Navarra, Navarra Health Service—Osasunbidea, Pamplona, Spain; 5 Research Unit AP-OSIS Gipuzkoa, IIS Biodonostia, San Sebastián, Spain; 6 San Juan Health Center, Navarra Health Service—Osasunbidea, Pamplona, Spain; 7 Frailty Day-Hospital, Gérontopôle de Toulouse, Department of Geriatric Medicine, CHU de Toulouse-Purpan, Toulouse, France; University of Brescia, ITALY

## Abstract

**Background/Objectives:**

The clinical status of older individuals with multimorbidity can be further complicated by concomitant geriatric syndromes. This study explores multimorbidity patterns, encompassing both chronic diseases and geriatric syndromes, in geriatric patients attended in an acute hospital setting.

**Design:**

Retrospective observational study.

**Setting:**

Unit of Social and Clinical Assessment (UVSS), Miguel Servet University Hospital (HUMS), Zaragoza (Spain). Year, 2011.

**Participants:**

A total of 924 hospitalized patients aged 65 years or older.

**Measurements:**

Data on patients’ clinical, functional, cognitive and social statuses were gathered through comprehensive geriatric assessments. To identify diseases and/or geriatric syndromes that cluster into patterns, an exploratory factor analysis was applied, stratifying by sex. The factors can be interpreted as multimorbidity patterns, i.e., diseases non-randomly associated with each other within the study population. The resulting patterns were clinically assessed by several physicians.

**Results:**

The mean age of the study population was 82.1 years (SD 7.2). Multimorbidity burden was lower in men under 80 years, but increased in those over 80. Immobility, urinary incontinence, hypertension, falls, dementia, cognitive decline, diabetes and arrhythmia were among the 10 most frequent health problems in both sexes, with prevalence rates above 20%. Four multimorbidity patterns were identified that were present in both sexes: Cardiovascular, Induced Dependency, Falls and Osteoarticular. The number of conditions comprising these patterns was similar in men and women.

**Conclusion:**

The existence of specific multimorbidity patterns in geriatric patients, such as the Induced Dependency and Falls patterns, may facilitate the early detection of vulnerability to stressors, thus helping to avoid negative health outcomes such as functional disability.

## Introduction

Multimorbidity, the presence of more than one chronic disease in a patient, affects more than half of the elderly population and almost all hospitalized geriatric patients [1]. Health systems do not specifically consider this population and their unique healthcare requirements [2].

The clinical care of older individuals can be complicated by a high frequency of concomitant geriatric syndromes. These syndromes consist of several conditions that are multifactorial in nature, rarely limited to a single organic system, and commonly associated with poor health outcomes [3]. The combination of multimorbidity and geriatric syndromes increases disability, mortality, and institutionalization rates [4].

Despite a growing emphasis on comprehensive care for chronic diseases, the organizational structure of current healthcare services is fragmented, with a strong emphasis on medical specializations [2]. Moreover, recommended management approaches to multimorbidity are lacking in most practice guidelines, which are the main scientific evidence-based tool available to clinicians [5]. Consequently, healthcare systems fail to appropriately address the healthcare needs of geriatric patients with multimorbidity. Inadequate clinical assessment of older patients leads to iatrogenesis, duplication of diagnostic and therapeutic interventions, and deviation from the aspects of healthcare that are most important to the patients themselves (e.g., pain elimination, preservation of functional and cognitive capacity, optimization of drug therapy) [6].

Recent studies have sought to identify systematic associations among diseases (i.e., associative multimorbidity), and have confirmed the existence of clinically plausible multimorbidity patterns that evolve over time [7]. Such beyond-chance associations among diseases may occur when one disease is directly responsible for others (i.e., complicating multimorbidity) or when several diseases share common or correlated risk factors (i.e., causal multimorbidity), which may be biological, socio-economic, cultural, environmental or behavioural in nature [8]. In both cases, the potential for secondary and tertiary prevention is high, underscoring the importance of these types of studies.

To date, the majority of studies of multimorbidity patterns have been limited to chronic diseases, so as to increase the likelihood of co-occurrence of conditions [9]. Despite their clinical relevance, very few studies have included geriatric syndromes in their analyses, probably due to the limited availability of relevant data in existing patient databases. In fact, the few studies that did study geriatric syndromes collected data either by comprehensive geriatric assessment [10,11] or patient self-reporting [4,12].

The present study explored multimorbidity patterns in geriatric patients attending an acute hospital, examining both chronic diseases and geriatric syndromes. Better knowledge of how these conditions cluster in older individuals could help clinicians and researchers better understand poor health outcomes in certain types of patients. Furthermore, our results may help guide the implementation of prevention strategies and the design of clinical practice guidelines adapted to the specific healthcare needs of this population group.

## Methods

### Study design, population and variables

This retrospective observational study included patients of 65 years or more who attended the Unit of Social and Clinical Assessment (UVSS) of the Miguel Servet University Hospital (HUMS) in Zaragoza (Spain) during 2011. This UVSS is located in a public tertiary hospital of the Aragon Health Service (SALUD) that serves a population of approximately 400,000 inhabitants. It consists of an interdisciplinary team (geriatrician, nurse, and social and administrative worker), one of whose main objective is to detect and evaluate recently hospitalized geriatric patients at risk of disability and dependency, and to minimize these risks.

The dataset included information on the clinical, functional, cognitive and social statuses of all attended patients. Functional assessment was conducted using the Barthel Index [13], and pre- and post-admission cognitive assessment was carried out using the nationally validated Red Cross Mental Scale (RCMS) [14] and Pfeiffer’s test [15], respectively. The diagnoses of each patient before and during hospitalization were grouped in Expanded Diagnosis Clusters (EDCs) using the ACG System. Only chronic EDCs included on a validated list of 115 EDCs published by Salisbury et al. [16] were considered. Information on the following geriatric syndromes was included in the database: immobility, urinary incontinence, constipation, pressure ulcers, cognitive decline, dementia, delirium, depression, falls, insomnia, visual impairment, hearing loss, malnutrition, dysphagia, and pain.

Immobility was defined as a decreased ability to perform activities of daily living due to impairment of motor functions. Urinary incontinence was defined as an objectively demonstrable involuntary loss of urine. Constipation was defined as less than two bowel movements per week. Pressure ulcers were defined according to the four grades established by the European Pressure Ulcer Advisory Panel (EPUAP) [17]. Cognitive impairment was assessed using Pfeiffer’s test, which was performed at the time of hospitalization, once a previous diagnosis of dementia and/or delirium had been ruled out. A previous diagnosis of dementia was based on the patient’s medical record data and was established using the RCMS. The ACG System included dementia and delirium in a single EDC category (NUR 11 “Dementia and delirium”). The criteria of the Diagnostic and Statistical Manual of Mental Disorders (DSM-IV-TR) [18] were used for the diagnosis of depression and insomnia. A fall was defined as the result of any event that caused the patient to end up on the ground against their will, according to the WHO definition [19]. Visual impairment and hearing loss were defined as self-reported difficulty hearing or seeing the interviewer without technical assistance. Malnutrition was defined as an intake that was less than 50% of the required daily allowance, as reported by the patient or caregiver, together with hypoalbuminemia and hypocholesterolemia. Pain was defined as any subjective complaint of an unpleasant sensory or emotional experience associated with actual or potential tissue damage, either acute or chronic. Dysphagia was defined as a difficulty swallowing liquids and/or solids that affected one or more phases of swallowing.

This study was approved by the Clinical Research Ethics Committee of Aragon (CEICA for its initials in Spanish). Patient written consent was not required as the study did not involve interventions on individuals, the use of human biological samples, or the analysis of personally identifiable data. The study involved the statistical analysis of anonymous data contained in previously existing databases and obtained with prior permission from the corresponding entity.

### Statistical analysis and clinical interpretation

A preliminary characterization of the population was performed by calculating the frequencies of demographic (i.e., age and sex), clinical (i.e., diseases/geriatric syndromes, functional and cognitive statuses) and utilization (i.e., polypharmacy, admissions, visits to the emergency room) variables. The prevalence rates of all chronic diseases and geriatric syndromes considered in the study were calculated separately for men and women (Table 1).

**Table 1:** Prevalence rates of chronic diseases and syndromes in the study population by sex.

Conditions	Overall	Men	Women
Immobility	89.39	86.72	91.43
Urinary incontinence	80.09	73.93	84.76
Falls	61.26	52.38	68.00
Hypertension	60.5	53.88	65.52
Cognitive decline	31.93	26.32	36.19
Dementia, delirium	30.19	28.57	31.43
Type 2 diabetes	24.03	24.81	23.43
Cardiac arrhythmia	22.08	26.57	18.67
Disorders of lipoid metabolism	21.97	22.06	21.90
Dysphagia	20.45	24.31	17.52
Constipation	19.37	18.80	19.81
Cerebrovascular disease	17.53	21.80	14.29
Vision impairment	17.32	14.79	19.24
Pressure ulcers	15.15	16.29	14.29
Arthropathy	14.29	10.53	17.14
COPD	14.18	26.07	5.14
Depression	12.55	9.52	14.86
Ischemic heart disease	12.01	16.29	8.76
Cataract, aphakia	11.36	10.53	12.00
Fractures (excluding hip fracture)	10.71	6.27	14.10
Hearing loss	9.96	9.77	10.10
Pain	9.52	10.53	8.76
Congestive heart failure	9.20	8.52	9.71
Hip fracture	8.23	4.51	11.05
Cardiovascular disorders, other	7.79	7.27	8.19
Cardiac valve disorders	7.36	8.52	6.48
Prostatic hypertrophy	7.25	16.29	0.00
Iron deficiency, other deficiency anaemias	6.93	6.27	7.43
Chronic renal failure	6.93	8.77	5.52
Thyroid disease	6.60	4.01	8.57
Osteoporosis	6.17	2.51	8.95
Malnutrition	4.22	5.76	3.05
Malignant neoplasms, prostate	4.11	9.52	0.00
Asthma	3.90	2.76	4.76
Diverticular disease of colon	3.57	4.51	2.86
Parkinson's disease	3.35	4.51	2.48
Obesity	3.35	2.26	4.19
Malignant neoplasms, colorectal	3.25	4.51	2.29
High impact malignant neoplasms	3.14	4.51	2.10
Glaucoma	3.03	2.76	3.24
Malignant neoplasms, breast	2.38	0.50	3.81
Anxiety	2.27	1.25	3.05
Tuberculosis infection	2.27	3.76	1.14
Cardiomyopathy	1.95	3.01	1.14
Seizure disorder	1.95	2.26	1.71
Acute myocardial infarction	1.84	2.76	1.14
Malignant neoplasms of the skin	1.84	1.50	2.10
Pulmonary embolism	1.84	0.75	2.67
Autoimmune and connective tissue diseases	1.84	1.00	2.48
Deep vein thrombosis	1.73	1.50	1.90
Sleep apnea	1.62	2.76	0.76
Respiratory disorders, other	1.62	1.00	2.10
Renal calculi	1.52	1.75	1.33
Malignant neoplasms, lung	1.52	2.51	0.76
Amputation	1.30	2.76	0.19
Malignant neoplasms, bladder	1.19	1.50	0.95
Peripheral neuropathy, neuritis	1.19	1.25	1.14
Chronic liver disease	1.08	0.75	1.33
Malignant neoplasms, liver and biliary tract	1.08	1.25	0.95

To analyse the clustering of diseases and/or geriatric syndromes into patterns, we employed an exploratory factor analysis, stratified by sex. This method identifies the tendencies of diseases to co-occur by selecting sets of variables with potentially common underlying causal factors. This approach thus provides results of etiological interest. The factors identified by this analysis can be interpreted as multimorbidity patterns, i.e., diseases that are non-randomly associated with one other within the study population (associative multimorbidity).

Factor analysis was performed using a tetrachoric correlation matrix to account for the dichotomous nature of the variables (i.e., presence/absence of a given condition) [20]. The use of the classical Pearson’s correlation coefficients for dichotomous variables results in mathematical artefacts, given the absence of a linear relationship among the variables and the restriction of the number of categories within one variable, which shrinks the magnitude of the correlations [21]. Factor extraction was performed using the principal factor method, and the number of factors extracted was determined using sedimentation graphs in which the eigenvalues of the correlation matrix were represented in descending order. The number of factors extracted corresponds to the sequence number of the eigenvalue that produces the inflection point of the curve.

To increase the epidemiological interest of the study, only health problems with prevalence rates >5% were included in each group of men and women. To identify the conditions that defined each multimorbidity pattern, those with scores ≥0.25 for each factor were selected (the same empirical threshold employed in previous studies [7,22,23]). Higher factor scores (i.e., closer to 1) indicate stronger associations between the condition and a given pattern. Conversely, if a given disease is relatively independent of a given factor, the resulting obtained score will be closer to 0.

The final phase of the analysis was intended to determine the clinical relevance of the patterns identified, and was conducted by five medical doctors (two geriatricians [MCS, NMV], two general practitioners [IVM, PAH], and one specialist in public health [APT]), first independently and then all together.

Statistical analyses were performed using STATA 12.0 software.

## Results

The total study population consisted of 924 patients, of whom 99.7% experienced multimorbidity and more than half (56.8%) were women. The mean age of the study population was 82.1 years (SD 7.2); almost 85% of the patients were over 74 years, and nearly 40% were at least 85 years old. The mean age of women was significantly higher than that of men (83.5 vs. 80.2 years, p<0.001).

**Fig 1:**
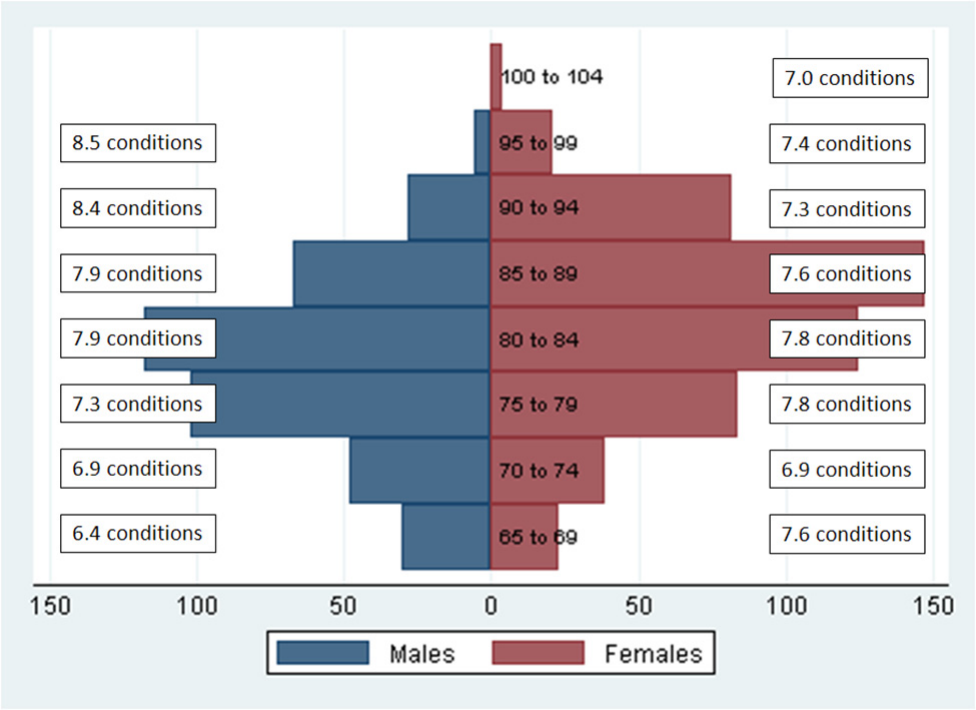
Mean number of conditions by sex and age group.

Multimorbidity was present in all age and sex groups (Fig 1). Compared with women of the corresponding age group, the multimorbidity burden was lower in men under 80 years but higher in those over 80 years. The level of multimorbidity in women between 65 and 69 years corresponded to that of men 15 years older.

Significant differences in functional and cognitive status were observed by sex; women showed higher rates of severe/total dependency both before and after hospitalization (Table 2). Men were more frequently on polymedication and showed higher rates of intense hospital use (p<0.05). Functional and cognitive status decreased after admission to hospital in both men and women.

**Table 2:** Main characteristics of the study population. ER: emergency room.

	Total (N = 924)	Men (n = 399)	Women (n = 525)	p-value*
**Pre-admission functional status** ^a^				
Independency-low dependency (%)	68.18	72.18	65.14	<0.05
Moderate dependency (%)	12.77	11.03	14.10	
Severe-total dependency (%)	15.15	12.03	17.52	
**Post-admission functional status** ^a^				
Independency-low dependency (%)	5.19	8.27	2.86	<0.001
Moderate dependency (%)	7.79	11.03	5.33	
Severe-total dependency (%)	84.31	77.19	89.71	
**Pre-admission cognitive status** ^b^				
Dementia (%)	16.88	11.53	20.95	<0.001
**Post-admission cognitive status** ^c^				
Cognitive decline (%)	31.93	26.32	36.19	<0.001
**Taking ≥6 drugs pre-admission (%)**	23.16	27.07	20.19	<0.05
**≥3 admissions in the previous year (%)**	8.01	11.53	5.33	<0.001
**≥5 visits to the ER in the previous year (%)**	8.01	12.28	4.76	<0.001

* Chi-square test

^a^ Barthel Index (Independency-low dependency: 60-100/100; Moderate dependency: 55-40/100; Severe-total dependency: ≤35/100)

^b^ Red Cross Mental Scale (Dementia: 4-5/5) and background of dementia in electronic clinical record

^c^ Pfeiffer’s Test (Cognitive decline: 3-10/10)

Immobility, urinary incontinence, hypertension, falls, dementia, cognitive decline, diabetes and arrhythmia were among the 10 most frequent health problems in both sexes, with prevalence rates above 20%. The next most frequent conditions were respiratory problems and dysphagia in men, and dyslipidaemia, constipation and vision impairment in women (Table 1).

Four different multimorbidity patterns were identified in the population, all of which were present in women and men (Tables 3 and 4). Out of the 31 health conditions included in the pattern identification process, 27 were common to men and women. Another two conditions were present exclusively in men (prostate malignant neoplasm and chronic obstructive pulmonary disease [COPD]) and two in women (osteoporosis and hip fracture). Only seven conditions (anaemia and hearing loss in men and constipation, dysphagia, pain, chronic renal failure and pressure ulcers in women) were present in more than one pattern.

**Table 3:** Factor scores of the conditions included in each pattern in men. KMO: 0.56. Percentage of cumulative variance: 37.90%. Factors score >0.25 are highlighted in bold.

Conditions	Cardiovascular pattern	Induced Dependency pattern	Falls pattern	Osteoarticular pattern
Immobility	0.1262	**0.7860**	-0.0402	-0.0879
Urinary incontinence	0.1189	**0.8500**	-0.0741	0.0716
Constipation	0.0619	0.1750	0.0341	**0.5628**
Cognitive decline	-0.0098	**0.6340**	0.1714	-0.0830
Falls	-0.0657	0.0742	**0.4058**	-0.3527
Malnutrition	-0.0574	0.1361	-0.8196	0.0370
Dysphagia	0.1539	**0.4496**	-0.0192	0.1666
Pain	0.0747	-0.0844	0.1253	**0.5358**
Ischemic heart disease	**0.2505**	-0.0950	-0.4535	-0.1125
Congestive heart failure	**0.6969**	0.0248	0.0209	-0.0807
Cardiac valve disorders	**0.5348**	0.1147	-0.1517	0.1034
Cardiac arrhythmia	**0.6115**	0.0649	0.0845	-0.0839
Disorders of lipoid metabolism	**0.2927**	-0.1548	-0.3540	-0.1160
Hypertension	**0.4649**	-0.0955	-0.1434	0.0007
Cardiovascular disorders, other	0.1535	0.0472	-0.3451	-0.2840
Type 2 diabetes	**0.2714**	-0.2500	0.0253	-0.3951
Cataract, aphakia	0.1189	-0.1396	0.0398	0.2160
Prostatic hypertrophy	0.0344	-0.0117	0.2261	-0.1848
Iron deficiency, other deficiency anaemias	**0.4460**	0.0102	**0.6895**	-0.0893
Malignant neoplasms, prostate	**0.3680**	0.1532	0.0616	0.1098
Fractures (excluding hip fracture)	-0.2338	-0.1243	0.1521	**0.4629**
Cerebrovascular disease	**0.3767**	0.0579	0.1291	0.1762
Chronic renal failure	**0.5885**	0.0587	-0.0014	-0.2318
COPD	**0.3220**	-0.4243	-0.0440	0.1829
Arthropathy	-0.1007	0.0714	-0.1092	**0.5008**
Pressure ulcers	0.0750	**0.4149**	-0.3115	-0.4111
Depression	0.1254	0.0376	-0.1220	**0.2866**
Vision impairment	0.1082	-0.0737	**0.7345**	0.2000
Hearing loss	-0.0965	**0.3406**	**0.3887**	-0.2689
Dementia, delirium	0.1000	**0.6460**	-0.0620	0.2163

**Table 4:** Factor scores of the conditions included in each pattern in women. KMO: 0.55. Percentage of cumulative variance: 35.32%. Factor scores >0.25 are highlighted in bold.

Conditions	Cardiovascular pattern	Induced Dependency pattern	Falls pattern	Osteoarticular pattern
Immobility	0.0193	**0.7464**	0.2484	-0.0677
Urinary incontinence	0.1716	**0.8646**	0.0361	-0.0159
Constipation	**0.3585**	-0.0658	**0.5878**	0.0901
Cognitive decline	-0.0969	**0.5048**	-0.1904	-0.4781
Falls	-0.0093	-0.0895	0.2186	-0.6836
Dysphagia	0.0811	**0.5313**	-0.1231	**0.2908**
Pain	0.2313	0.1556	**0.5153**	**0.4888**
Ischemic heart disease	**0.3631**	-0.1772	-0.2275	-0.0655
Congestive heart failure	**0.8076**	0.1157	0.2147	-0.0427
Cardiac valve disorders	**0.4901**	-0.2304	-0.1382	-0.2384
Cardiac arrhythmia	**0.7782**	0.0639	0.0276	-0.0877
Disorders of lipoid metabolism	**0.2679**	-0.1448	-0.0345	0.2287
Hypertension	**0.4698**	-0.0643	-0.1024	0.1761
Cardiovascular disorders, other	**0.3730**	0.0328	0.1144	0.0160
Osteoporosis	-0.2056	-0.2239	**0.3868**	0.2050
Thyroid disease	0.1837	-0.2384	-0.3029	0.0851
Type 2 diabetes	0.2127	-0.0137	-0.3483	0.0060
Cataract, aphakia	0.0940	-0.3730	-0.0521	-0.1053
Iron deficiency, other deficiency anaemias	0.2249	-0.0690	-0.0053	**0.3674**
Fractures (excluding hip fracture)	-0.1989	0.0711	-0.0341	**0.3315**
Hip fracture	-0.0573	0.1560	**0.4478**	-0.1689
Cerebrovascular disease	0.2493	**0.2622**	-0.1408	-0.1955
Chronic renal failure	**0.5195**	**0.3864**	0.0089	0.2377
Arthropathy	-0.0229	-0.0208	0.0250	**0.4922**
Pressure ulcers	0.0123	**0.4574**	-0.4967	**0.3890**
Depression	-0.0510	0.0403	**0.3125**	0.1937
Vision impairment	0.1041	-0.2325	**0.4109**	-0.0692
Hearing loss	0.1328	-0.0274	**0.3889**	-0.0851
Dementia, delirium	-0.1013	**0.5430**	-0.1972	-0.2104

The first pattern, i.e., Cardiovascular pattern, grouped risk factors, such as hypertension, dyslipidaemia and type II diabetes (factor score close to the cut-off point in women: 0.21) together with other cardiovascular conditions such as valve disorders, arrhythmia, anaemia (factor score close to the cut-off point in women: 0.22), ischemic heart disease, congestive heart failure, cerebrovascular disease and chronic renal failure. Other cardiovascular disorders and constipation were exclusively present among women. In men, COPD and prostate cancer were also present in this pattern.

The second pattern, i.e., Induced Dependency pattern, consisted of a constellation of geriatric syndromes, most of which were common to both sexes (immobility, urinary incontinence, dysphagia, pressure ulcers, cognitive decline and dementia/delirium). Hearing loss was present in men only, and cerebrovascular disease and renal failure in women only.

The third pattern, i.e., Falls pattern, consisted of sensory deficits (i.e., vision and hearing), anaemia and falls in men, and sensory deficits, osteoporosis, hip fracture, depression, pain and constipation in women. Falls in women were close to the cut-off point (factor score: 0.22).

The last pattern, i.e., Osteoarticular pattern, differed between sexes. Arthropathy, fractures (other than hip fractures), and pain were common to both sexes, while depression and constipation were observed exclusively in men and anaemia, dysphagia and pressure ulcers in women only.

## Discussion

This study, which detected multimorbidity in almost all patients analysed, used a combined clinical and statistical approach to identify four clinically consistent multimorbidity patterns; Cardiovascular, Induced Dependency, Falls and Osteoarticular. In each pattern, coexistence of chronic diseases and geriatric syndromes was observed.

### Cardiovascular pattern

This pattern has been extensively described in the literature [24]. Its composition was highly consistent with the pathophysiology of the cardiometabolic syndrome, corroborating the clinical picture already described in older populations [7]. The demonstrated association between cardiovascular disease and COPD [25] and the high prevalence of the latter in men may explain their co-existence in this pattern. Prostate cancer was another non-cardiovascular condition included in this pattern. The potential adverse cardiovascular effects of antiandrogens used to treat prostate cancer patients remain the subject of much debate [26,27]. However, acknowledgment of these potential adverse effects is important given that the majority of these patients die from conditions other than cancer.

### Induced Dependency pattern

The coexistence in this pattern of the most prevalent geriatric syndromes (immobility and urinary incontinence) together with delirium and pressure ulcers appears to be related to the effects of common stressors, such as acute conditions and hospitalization in vulnerable or frail patients. Moreover, the presence of dementia in both sexes and the association with cerebrovascular disease and renal failure in women could further contribute to an increased risk of dependency in older patients. All of these conditions are frequently the consequence of previously existing diseases, which trigger further deterioration of health status. Incomplete assessment of existing geriatric syndromes and failure to address the underlying causes are known to give rise to new health problems [28]. This geriatric or frailty cascade may result in the development of new syndromes, avoidable cognitive or functional decline, or even death of the patient [29,30]. A systematic review by Tinetti et al. found that advanced age, cognitive and functional decline, and mobility impairment increased the risk of pressure ulcers, urinary incontinence, falls and delirium [31], a scenario that is in good agreement with our results. Previous studies have also revealed the potential negative effects of hospital admission, particularly deterioration towards dependency [32,33].

### Falls pattern

It should be noted that falls did not cluster with the rest of the syndromes included in the Induced Dependency pattern, perhaps due to the importance of this syndrome on its own in the elderly. Falls are the leading cause of injury in community-dwelling adults over 65 years [34]; between 30% and 40% of these individuals fall at least once per year [35], with severe consequences such as disability, institutionalization, or death [36]. In men, who have a lower prevalence of osteoporosis, falls are not usually associated with serious injuries. In women, falls, together with osteoporosis, are risk factors for hip fracture, which is up to three times more frequent in women [37].

Osteoporotic frailty fractures can cause substantial pain, and one of the side effects of the analgesic opiate derivatives used to treat this condition is constipation [38], also present in this pattern, although it can be caused by immobility as well [39]. Moreover, the severe and abrupt disabilities associated with hip fractures often lead to a decreased quality of life in older women, which may explain the inclusion of depression in this pattern [40]. Depression is also a recognized risk factor for falls and fractures in the elderly [41]. Vision and hearing impairment are also well-known factors that contribute to falls [42]. In fact, vision impairment alone has been associated with almost half of all falls in the older population [43].

### Osteoarticular pattern

Arthropathy, which has a high prevalence that increases with age, progresses with pain and functional limitation, requiring high doses of opioids [44]. Therefore, it is likely that the constipation present in men is due to opioid treatment [38] or immobility, also in this pattern. However, we were unable to explain the absence of an association between arthropathy and constipation in women, given the coexistence of pain in both sexes.

As in the Falls pattern, the consequences of the conditions comprising the Osteoarticular pattern appeared to be more severe in women, who were also affected by pressure ulcers, possibly caused by immobility. However, it should be noted that immobility syndrome per se did not feature in this pattern, an observation for which we have no plausible explanation.

### Comparison with other studies

The Cardiovascular pattern has been repeatedly described in previous studies [10–12,22,23,45,46]. This pattern is characterized by the co-occurrence of risk factors, cardiovascular diseases, metabolic disorders [10,22], cerebrovascular disease [10,12,22,23] and COPD (occasionally) [10,22,46].

For the other three patterns, similarities with previous studies were harder to find due to the limited number and types of diseases and/or geriatric syndromes considered by other researchers. For example, we were unable to corroborate the connection we observed between falls and sensory deficits because the former has never been included in studies of multimorbidity patterns. While our Osteoarticular pattern resembled the Anxiety, Depression, Somatoform disorders and Pain pattern described by Schäfer et al. [22], the association with fractures found in our study could not be compared as this condition was excluded from their analysis.

To the best of our knowledge, this is the first study to describe an Induced Dependency pattern, probably because no other studies of multimorbidity patterns have considered such a wide range of geriatric syndromes. Indeed, most previous studies included no more than three syndromes from the following list of conditions: hearing loss [10–12,22], visual impairment [10–12], urinary incontinence [4,22,46], falls [4,46], and insomnia [22]. Nevertheless, the need to fully explore the pathways that link chronic diseases and geriatric syndromes has been emphasized by some of these authors [4].

### Strengths and weaknesses

One of the main strengths of this study is its comprehensiveness in terms of the range of conditions considered in individual patients. Clinical complexity is addressed by including subjective symptoms and important geriatric syndromes, such as falls and cognitive impairment [47,48], rather than simply relying on disease counts. For this reason, we believe our statistical model is adequately adjusted for confounding diagnoses that may bias the results. Moreover, the idiosyncrasy of the UVSS ensures that all conditions are clinically verified by a trained professional, which increases the reliability of the diagnostic data. Factor analysis provides an overall picture of the associations between diseases in a given population without needing to establish an a priori list of conditions. The main advantage of this type of analysis is that unlike other multivariate statistical techniques, such as cluster analysis, diseases can form part of one or several patterns [24].

Among the limitations of the present study is the possible underreporting of some diseases/conditions. One such example is malnutrition, for which no objective nutritional assessment tool was available at the time of the study. Furthermore, we were unable to adjust by disease severity, which may have affected disease clustering. Because this study was cross-sectional, it is not possible to know when the various diseases and conditions analysed were incorporated into a given pattern. An etiological approach to the study of disease associations inevitably requires a longitudinal research design, which we propose as a future line of research. Finally, as only chronic diseases were considered for analysis, we were unable to study the role of acute conditions in the associative pathway between chronic diseases and/or geriatric syndromes. This may be especially relevant in the case of the Induced Dependency pattern, as acute conditions often trigger hospital admission and, indirectly, the frailty cascade.

### Clinical relevance of the findings

This study corroborated the existence of clinically consistent multimorbidity patterns among the geriatric population. Of particular interest was the Induced Dependency pattern, which consisted of geriatric syndromes that were not present in any of the other three patterns identified, and was largely similar for both sexes. This pattern could serve as an alarm signal in hospitalized geriatric inpatients and allow the identification of situations of vulnerability to stressors, such as hospitalization itself. Early detection of this signal would facilitate the prevention of negative health outcomes, such as functional disability. Comprehensive geriatric assessment and treatment are key interventions aimed at halting the aforementioned frailty cascade [49].

Another important finding of this study is the differences observed between sexes within the Falls pattern. The aggravating role of osteoporosis in women, as suggested by our results, necessitates greater prevention and treatment efforts to tackle this condition, especially on the part of primary care professionals. A greater focus on healthy lifestyles, strategies to actively prevent falls and early diagnosis of osteoporosis could help to improve the quality of life of these patients.

### Conclusions and future research

Our simultaneous analysis of diseases and geriatric syndromes in the older population revealed four clinically consistent multimorbidity patterns. One such pattern (i.e., Induced Dependency pattern), composed exclusively of geriatric syndromes, may act as a trigger of functional decline, underscoring the importance of global assessment of geriatric patients. Future longitudinal studies should investigate the effects of these patterns on specific health outcomes and their evolution over time in order to predict healthcare needs and, where possible, avoid negative health outcomes in patients in whom frailty is irreversible. Moreover, rather than considering one condition at a time, care guidelines and quality indicators should be designed to provide comprehensive and coordinated management of co-occurring diseases and geriatric syndromes.
